# Outcome of infections due to pandrug-resistant (PDR) Gram-negative bacteria

**DOI:** 10.1186/1471-2334-5-24

**Published:** 2005-04-08

**Authors:** Matthew E Falagas, Ioannis A Bliziotis, Sofia K Kasiakou, George Samonis, Panayiota Athanassopoulou, Argyris Michalopoulos

**Affiliations:** 1 Alfa Institute of Biomedical Sciences (AIBS), Athens, Greece; 2 Tufts University School of Medicine, Boston, Massachusetts, USA; 3 Alfa HealthCare, Athens, Greece; 4 Department of Medicine, University Hospital, University of Crete, School of Medicine, Heraklion, Greece; 5 Intensive Care Unit, "Henry Dunant" Hospital, Athens, Greece

## Abstract

**Background:**

The increasing problem of infections due to multidrug-resistant Gram-negative bacteria has led to re-use of polymyxins in several countries. However, there are already clinical isolates of Gram-negative bacteria that are resistant to all available antibiotics, including polymyxins.

**Methods:**

We present a case series of patients with infections due to pathogens resistant to all antimicrobial agents tested, including polymyxins. An isolate was defined as pandrug-resistant (PDR) if it exhibited resistance to all 7 anti-pseudomonal antimicrobial agents, i.e. antipseudomonal penicillins, cephalosporins, carbapenems, monobactams, quinolones, aminoglycosides, and polymyxins.

**Results:**

Clinical cure of the infection due to pandrug-resistant (PDR) Gram-negative bacteria, namely *Pseudomonas aeruginosa *or *Klebsiella pneumoniae *was observed in 4 out of 6 patients with combination of colistin and beta lactam antibiotics.

**Conclusion:**

Colistin, in combination with beta lactam antibiotics, may be a useful agent for the management of pandrug-resistant Gram-negative bacterial infections. The re-use of polymyxins, an old class of antibiotics, should be done with caution in an attempt to delay the rate of development of pandrug-resistant Gram-negative bacterial infections.

## Background

The incidence of multiresistant bacterial infections increases worldwide. This made the medical community to re-evaluate older agents such as colistin, for infections due to Gram-negative multiresistant bacteria, including *Pseudomonas aeruginosa*, *Klebsiella pneumoniae*, and *Acinetobacter baumannii*. Colistin was used for about two decades after its discovery in 1950, but the reported nephrotoxicity and neurotoxicity led to gradual decrease of its use. However, the antibiotic recently regained some popularity in several countries as a salvage antimicrobial agent against bacteria resistant to all other classes of antibiotics. Fortunately, several studies during the last five years did not verify the high rates of toxicity of the medication reported in the old literature [1,2].

The reuse of colistin is associated with a possible significant therapeutical problem, namely the advent of bacteria resistant to all classes of available antimicrobial agents, including the polymyxins. These bacteria are really pandrug-resistant. It should be noted that the definition of pandrug-resistant Gram-negative bacteria does not include always testing for colistin in many countries. For example, a high mortality rate (60%) was reported in a study of *Acinetobacter baumannii *infections from Taiwan; however, no colistin was used in the in vitro susceptibility testing and, most important, the drug was not given to patients [3,4]. There are recent reports from patients with cystic fibrosis with pandrug-resistant Gram-negative bacterial infections [5,6]. In addition, microbiological data about clinical isolates of Gram-negative bacteria resistant to colistin were included in some recent reports [7-12]. However, no study up to now has presented the characteristics of such patients, clinical information about the infections as well as the outcomes. The objective of this study was to present data regarding the clinical characteristics, therapeutic management, and clinical outcome of a group of patients with infections caused by pandrug-resistant (PDR) Gram-negative microorganisms.

## Methods

### Design of the study – data collection

Patients with infections caused by pandrug-resistant Gram-negative bacteria (resistant to all tested antibiotics, including colistin), who were hospitalized in the intensive care unit, during the period from 1/October/2000 to 31/August/2004 (hospital 1) were identified by the ICU's electronic database, and during the same period in hospital 2. The medical records of these patients were reviewed. Data for several variables, including demographic and clinical information, as well as results of laboratory and imaging tests of the patients were collected using a specially designed case report form and entered in a computer database.

### Definitions

Multidrug-resistant (MDR) *Acinetobacter baumannii*, *Pseudomonas aeruginosa*, or *Klebsiella pneumoniae *strains were defined if resistance of the isolates was observed to 5 out of the 7 anti-pseudomonal classes of antimicrobial agents, i.e. antipseudomonal penicillins, cephalosporins, carbapenems, monobactams, quinolones, aminoglycosides, and colistin. Clinically important subsets of MDR Gram-negative isolates are those sensitive only to colistin and carbapenems (CCOS-colistin-carbapenem-only-sensitive). An isolate was defined as colistin-only-sensitive (COS) if it was resistant to all anti-pseudomonal agents, except colistin, and as PDR if it exhibited resistant to all 7 anti-pseudomonal antimicrobial agents, including colistin. Intermediate sensitivity was considered as resistance.

The definition of the site of the infection and of the responsible pathogen were based on the clinical symptoms and signs of individual patients, imaging findings, and on the isolation of a possible pathogen from evaluable clinical specimens. Specifically, diagnosis of pneumonia required two or more serial chest radiographs with at least one of the following: new or progressive and persistent infiltrate, consolidation, cavitation, or pleural effusion. In addition, patients must have had fever >38°C with no other recognized cause, or abnormal white blood cell count [leukopenia (< 4000 WBC/mm^3^) or leukocytosis (≥ 12.000 WBC/mm^3^)], and at least two of the following: new onset of purulent sputum or change in character of sputum, increased respiratory secretions or increased suctioning requirements, new onset or worsening of cough or dyspnea or tachypnea, rales or bronchial breath sounds, or worsening gas exchange [13].

Bacteremia required either growth of a recognized pathogen from one or more blood specimen cultures or at least one of the following signs or symptoms: fever (>38°C), chills, or hypotension *and *at least one of the following: a) common skin contaminant (e.g., diphtheroids, *Bacillus *sp., *Propionibacterium *sp., coagulase-negative staphylococci, or micrococci) grown from two or more blood cultures drawn on separate occasions or b) common skin contaminant (e.g., diphtheroids, *Bacillus *sp., *Propionibacterium *sp., coagulase-negative staphylococci, or micrococci) grown from at least one blood culture from a patient with an intravascular line and physician- instituted antimicrobial therapy [13].

Infections at other body sites or fluids, such as urinary tract infections, meningitis, and central venous catheter-related infections were defined based on guidelines from the Centers for Disease Control and Prevention [13].

### Microbiological testing

Identification of all causative microorganisms was performed by classic microbiologic methods. Susceptibility testing was performed both by the disk diffusion method and according to an automated broth microdilution method (bioMerieux, Vitek II, Hazelwood, MO). The breakpoints were those defined by the Clinical and Laboratory Standards Institute (CLSI) [14]. The MIC breakpoint used to identify bacteria susceptible to colistin was 2 mg/l. Bacteria for which MIC was 2 mg/l or less were considered susceptible while bacteria with MIC 4 mg/l or more were considered resistant. Susceptibility to colistin was tested by means of the disk diffusion method with the use of 10 μg of colistin sulfate disk (Oxoid, Basingstoke, Hants, England). Isolates were considered sensitive if the inhibition zone was 11 mm or more. The results were verified by the E-test technique using the same cut points to determine the resistant isolates (sensitive: 2 mg/l or less, resistant: 4 mg/l or more). No molecular biology testing was performed to clarify whether isolates of the same microbial species represented the same or different strains.

## Results

Seven patients were identified to have infection due to pandrug-resistant Gram-negative bacteria during the study periods. The isolated organisms were found to be resistant to the following antibiotics; amikacin, aztreonam, cefepime, ceftazidime, ciprofloxacin, gentamicin, imipenem, meropenem, netilmicin, pefloxacin, piperacillin/tazobactam, ticarcillin/clavulanic acid, trimethoprim/sulfamethoxazole, cefpirome, colistin, and isepamicin. The clinical features of patients, including the isolated pandrug-resistant strains, the antimicrobial agents used for the management of the infections, as well as their outcome, are shown in the table. The colistin formulation that was administered to the patients was sodium colistimethate (Colomycin, Forest Laboratories^®^, Kent, UK). One milligram of the colistin used is approximately equal to 12,500 IU. Other data related to the hospitalization of these patients are summarized below.

### Patient 1

A 47-year-old male was transferred from another ICU due to multiple chest injuries after a car accident, respiratory tract infection, and ICU polyneuropathy. Based on clinical and microbiological evidence, therapy for severe pneumonia was initiated on the 2^nd ^day of the ICU stay, with a combination of imipenem/cilastatin, gentamicin, ofloxacin and vancomycin that were given for 10, 22, 6 and 14 days respectively. Imipenem/cilastatin was substituted with piperacillin/tazobactam given for 12 days, and piperacillin/tazobactam was then substituted with ceftriaxone given for 10 days. On the 30^th ^day teicoplanin was added to the regimen, given for 21 days. On the 40^th ^day of the ICU stay, a PDR *Pseudomonas aeruginosa *strain was isolated from his bronchial secretions. He was subsequently treated, due to the persistence of the infection, with a prolonged course of intravenous colistin for a total of 52 days, combined with imipenem/cilastatin for 12 days, then meropenem for 23 days and then ofloxacin for 16 days. On the 54^th ^day a strain of *Pseudomonas aeruginosa *sensitive only to colistin (COS) was isolated from his bronchial secretions. The infection was gradually controlled and, finally, the patient was discharged from the hospital in a good condition.

### Patient 2

A 47-year-old female was admitted to the ICU due to possible meningitis. She had been operated for meningioma and had a ventriculo-peritoneal shunt. The shunt was removed at the day of admission with simultaneous insertion of external cerebrospinal fluid drainage. The initial cultures of CSF specimens were negative. The patient was treated with intravenous ceftriaxone and vancomycin. A CSF specimen, taken on the 24^th ^day of ICU stay, grew a MDR *Pseudomonas aeruginosa *strain (sensitive only to colistin and piperacillin/tazobactam, intermediate sensitive to imipenem and meropenem) and ceftriaxone was substituted with piperacillin/tazobactam and meropenem. Due to deterioration of her clinical condition several combinations of antimicrobial agents were subsequently given, including ciprofloxacin for 19 days, chloramphenicol for 11 days, colistin for 23 days, and gentamicin for 6 days. On the 62^nd ^day of the ICU stay, a PDR *Pseudomonas aeruginosa *strain was isolated from a cerebrospinal fluid specimen. Until the isolation of the PDR strain, repeated CSF specimen cultures grew MDR *Pseudomonas aeruginosa*. A gradual change in the *Pseudomonas *susceptibility was observed from multidrug-resistance to pandrug-resistance. The patient died on the 66^th ^day of hospitalisation due to *Pseudomonas *meningitis.

### Patient 3

An 18-year-old male was admitted to the ICU with fractures of the 2^nd ^and 3^rd ^cervical vertebrae and concomitant acute respiratory failure type I. Pneumonia was diagnosed clinically and confirmed microbiologically. *Staphylococcus aureus *and COS *Acinetobacter baumannii *were repeatedly isolated from bronchial secretions during the second and the third week of his hospitalization, respectively. Different combinations of antibiotics were provided, due to the persistence of severe pneumonia. The antibiotics were vancomycin, broad-spectrum cephalosporins, clindamycin, piperacillin/tazobactam, aminoglycosides, quinolones, meropenem and colistin (given for a total of 21, 15, 9, 7, 25, 7, 6 and 11 days respectively, up to the 34^th ^day of ICU stay). On the 34^th ^day of ICU stay PDR *Pseudomonas aeruginosa *grew from his bronchial secretions. At that time, he was receiving colistin, meropenem, ofloxacin and gentamicin. He continued to receive the same antimicrobial combination. PDR *Pseudomonas aeruginosa *was not isolated again from subsequent cultures of bronchial secretions. Instead, *Acinetobacter baumannii *grew again from bronchial secretions on the 35^th ^day of hospitalization and in subsequent cultures by the 45^th ^day of ICU stay. Meanwhile a gradual improvement of the infection was noted. The patient was transferred to a specialized orthopedic center after 78 days of ICU stay, in good general condition, without evidence of pneumonia or other infection.

### Patient 4

A 72-year-old female was admitted to the ICU with a complicated, severe urinary tract infection (UTI) due to COS *Pseudomonas aeruginosa *and acute respiratory distress syndrome (ARDS). During the last trimester, she had been hospitalized twice for episodes of UTIs. Treatment with intravenous colistin and meropenem was initiated on ICU admission and 15 days later urine specimen cultures were negative. However, due to persistence of fever the treatment was continued. Vancomycin was added to the regimen after isolation of *Enterococcus faecium *from cultures of the tip of the central venous catheter (CVC) on the 8^th ^day of ICU stay. On the 33^rd ^day, PDR *Klebsiella pneumoniae *was isolated from the tip of the CVC. Due to unavailability of antimicrobial agents with *in vitro *effect against the pathogen, no change in the antimicrobial regimen was performed. Her clinical condition was complicated on the 37^th ^day with lower respiratory tract infection and bacteremia due to COS *Pseudomonas aeruginosa*, for which a short course of gentamicin was added to the treatment. From the tip of the CVC, MDR *Klebsiella pneumoniae *resistant to all tested drugs (not tested for colistin) was isolated again on the 48^th ^day, and COS *Klebsiella pneumoniae *on the 63^rd ^day of ICU stay. Her clinical condition improved gradually, although COS *Pseudomonas aeruginosa *kept being isolated in cultures of bronchial secretions up to the 78^th ^day of ICU stay. She was transferred in good condition to another hospital for continuation of her care, without fever or other clinical and laboratory evidence of infection, after 85 days of ICU stay.

### Patient 5

A 56-year-old male was admitted to the ICU after elective operation for a thoraco-abdominal aortic aneurysm and intra-operative hemorrhage. On the 24^th ^day of ICU stay the patient was re-operated for infection of the thoracotomy with pus collection, which required drainage. On the 52^nd ^day PDR *Klebsiella pneumoniae *was isolated from bronchial secretions. He had already received 31 days of colistin treatment, combined with ampicillin/sulbactam (for 22 days) and trimethoprim/sulphamethoxazole (for 8 days), all given for treatment of polymicrobial pneumonia caused by MDR strains of *Acinetobacter baumannii *and *Klebsiella pneumoniae *and bacteremia by MDR *Klebsiella pneumoniae *strain. By the time of isolation of the PDR microorganism the patient's condition was steadily deteriorating; eventually irreversible septic shock and multiple organ failure led to his death on the 68^th ^day of ICU stay.

### Patient 6

A 35-year-old male was transferred to our hospital from another medical center with infection of the lower respiratory tract and UTI due to COS *Pseudomonas aeruginosa*. The patient had a history of rupture of an aneurysm of the circle of Willis that was surgically managed. During his prolonged hospitalization, episodes of obstructive hydrocephalus and CNS infection complicated his clinical condition, which necessitated admission to the ICU. On the 32^nd ^day of ICU stay, PDR *Pseudomonas aeruginosa *was isolated from a urine specimen. By that time, he had received several combinations of antimicrobial agents, including 6 days of treatment with colistin, for lower respiratory tract infection with COS *Pseudomonas aeruginosa*. After isolation of the PDR pathogen he received combination treatment with colistin and ceftazidime leading to clinical cure of the UTI. The clinical condition of the patient gradually improved and he became afebrile. However, the patient's respiratory tract remained constantly colonized with MDR and COS *Pseudomonas aeruginosa *strains. He was transferred to the neurosurgical ward after 134 days of ICU stay, in improved condition. Thirty days later, pneumonia relapsed and progressed into septic shock and multiple organ failure, and subsequent death.

### Patient 7

An 82-year-old male was admitted in the nephrology clinic due to cellulitis of the right lower limb. The patient had a history of chronic renal failure and a recent episode of bronchiolitis obliterans organizing pneumonia (BOOP). Blood specimen cultures performed on admission and 24 hours later, grew both PDR *Pseudomonas aeruginosa *strain. Treatment with intravenous piperacillin/tazobactam was initiated. A quick improvement of the cellulitis was noted. The therapeutic regimen was not changed when a result for isolation of a PDR *Pseudomonas aeruginosa *strain from the blood cultures became available. The patient was discharged to home after 10 days of treatment, with the cellulitis cured. In addition, subsequent blood cultures did not reveal any isolates.

## Discussion

We present the first case series that focuses on clinical information and outcome of infections due to pandrug-resistant Gram-negative bacteria. Our study shows that the isolation of a pandrug-resistant Gram-negative rod from clinical specimens does not necessarily mean a bad outcome. This may be explained by several reasons. First, the achieved concentration of several antimicrobial agents in some body fluids, including the urine may exceed the minimal inhibitory concentration of the isolated pathogen, even if the in vitro susceptibility result would place the isolate in the resistant category. Second, infections, even severe, sometimes are self-limited without the use of antimicrobial agents, as the pre-antibiotic era taught us. Third, pandrug-resistant bacteria may be colonizers of the respiratory tract in patients receiving several classes of antimicrobial agents for a long period of time, while the real pathogen may not be isolated. Fourth, it has been shown that occasionally MDR organisms may exhibit decreased virulence compared to other more sensitive organisms of the same species.

In addition, some of our patients received combinations of colistin with antimicrobial agents such as carbapenems, third generation cephalosporins, and quinolones. This fact represents an important practical observation of our study. Specifically, 4 patients achieved complete cure from the pandrug-resistant Gram-negative bacterial infection, with combinations of colistin with other antimicrobial agents, with which may have a synergistic effect. Previous experimental and clinical studies showed promising results regarding the synergistic effect of colistin with antibiotics from other classes such as β-lactams (piperacillin, ceftazidime, aztreonam, meropenem), aminoglycosides (amikacin, gentamicin), sulfonamides (trimethoprim/sulfamethoxazole), rifamycins (rifampicin), and quinolones (ciprofloxacin) against MDR Gram-negative bacteria [15-17]. However, clinical trials are needed to evaluate the in vivo results of these combinations.

Colistin resistance among Gram-negative organisms has been reported up today in some in vitro studies, as well as in surveillance studies for antimicrobial resistance [7,8,18]. In most of these studies the isolated strains of *Pseudomonas aeruginosa*, *Klebsiella pneumoniae*, and *Acinetobacter baumannii *exhibited a greater than 98% susceptibility to colistin or polymyxin B. However, reports about clinical parameters and outcome of infections due to colistin-resistant Gram-negative bacteria are rare in the literature. A search of PubMed and Current Contents databases revealed only 2 such reports. Both of them referred to cystic fibrosis patients [5,6]. In the first study, 5 out of 44 adult patients with cystic fibrosis and *Pseudomonas aeruginosa *chronic bronchial infection developed resistance to colistin. In the second study, colistin resistant *Pseudomonas aeruginosa *was found in 5 out of 150 children with cystic fibrosis over a 5-year period of follow up. Our study confirms the rarity in the incidence of such infections since in a study period of several years we recorded only seven such infections in two hospitals.

Gram-negative bacteria can develop resistance to polymyxins through mutation or adaptation mechanisms [19]. Mutation is inherited, low-level, and independent of the continuous presence of the antibiotic, whereas adaptation is the opposite (non-inherited, high level, and requires the continuous presence of the antibiotic). Almost complete cross-resistance exists between colistin and polymyxin B [20,21]. It has been suggested that a basic mechanism of resistance in *Pseudomonas aeruginosa *strains is that the outer membrane protein OprH blocks the self-promoted uptake pathway of polymyxins by replacing Mg^2+ ^on the LPS molecule. Thus, the overexpression of OprH caused by mutation or as a result of adaptation to an Mg^2+^-deficient medium can be associated with resistance to polymyxins [22,23].

Studies on polymyxin-resistant *Pseudomonas aeruginosa *strains have suggested that cell surface changes are related with the development of resistance. These include alterations of the outer membrane of the bacteria cell (reduction of LPS, reduced levels of specific outer membrane proteins, reduction in cell envelope Mg^2+ ^and Ca^2+ ^contents, and lipid alterations). Specifically, the absence of 2-hydroxylaurate, the presence of 4-aminoarabinose, and increase in the palmitate content of lipid A has been associated with resistance of *Pseudomonas aeruginosa *to polymyxins [5,24]. In addition, studies in *Salmonella *spp. have shown that the two component systems PhoP-PhoQ and PmrA-PmrB regulate polymyxin resistance. The transcription of PmrA-PmrB is activated by the PhoP-PhoQ or by growth in mild acidic conditions (pH = 5.8) and micromolar concentrations of Mg^2+ ^(10 μM). The products of PmrA-PmrB activated genes are responsible for the modifications on the LPS molecules of Gram-negative bacteria. Consequently, the affinity of polymyxins to the bacterial cell surface is reduced [24].

In addition, a recent study in *Yersinia *spp. demonstrated that an efflux pump/potassium antiporter system may be associated with the mediation of resistance to cationic antimicrobial peptides in general, including polymyxin B [25]. Although enzymatic resistance of bacteria to polymyxins has not been reported, it is interesting that *Bacillus polymyxa *subsp.*colistinus *produces colistinase that inactivates colistin [26].

Guidelines from Clinical and Laboratory Standards Institute (CLSI) the about the in vitro determination of the minimum inhibitory concentrations (MICs) of different microorganisms to colistin using broth dilution and agar dilution techniques were established in 1970. However, due to the rare use of colistin in the United States and most countries, the CLSI guidelines were not modified after 1981 and were withdrawn in 2000. The re-use of polymyxins during the last years will make necessary the re-evaluation of the susceptibility breakpoints. A common method for susceptibility testing of colistin has been the disk diffusion method that uses 10 micrograms of colistin sulfate disk (Oxoid, Basingstoke, Hants, England). Isolates are considered sensitive if the inhibition zone is ≥ 11 mm. It is important to emphasize that in clinical practice it is colistimethate sodium, not colistin sulfate that is used for intravenous administration. There is generally agreement in the results obtained from agar dilution and broth microdilution methods regarding testing of colistin sulfate [27]. However, it has been suggested that the disk diffusion test should be confirmed with a dilution method, because the disk diffusion method may reveal false-susceptible microorganisms [27].

We should acknowledge several limitations of this study. First, molecular typing was not performed thus the possibility that PDR isolates derived from the previous isolated microorganisms cannot be determined. Second, MDR or PDR bacteria were markers of the patients' condition and the effect of the infection and of the treatment on the outcome of the patients cannot be adequately evaluated.

## Conclusion

In an era of alarmingly increasing bacterial resistance, our study adds clinical information about the outcome of infections due to pandrug-resistant Gram-negative bacteria. Further work is needed to elucidate the possible in vitro synergistic effect of combinations of colistin with antibiotics from several classes and their effectiveness in clinical practice.

## Competing interests

The author(s) declare that they have no competing interests.

## Authors' Contributions

MEF conceived the idea for the study; IAB, SKK and PA collected the data; MEF drafted the manuscript. All authors contributed in the writing and preparation of the manuscript. All authors read and approved the final manuscript.

**Table 1:** Characteristics of the reported patients and outcomes of the infections.

**Patient no.**	**1**	**2**	**3**	**4**	**5**	**6**	**7**
**Isolated organism**	*Pseudomonas aeruginosa*	*Pseudomonas aeruginosa*	*Pseudomonas aeruginosa*	*Klebsiella pneumoniae*	*Klebsiella pneumoniae*	*Pseudomonas aeruginosa*	*Pseudomonas aeruginosa*
**Site of isolation**	Bronchial secretions	Cerebrospinal fluid	Bronchial secretions	Tip of central venous catheter	Bronchial secretions	Urine	Blood
**Date of isolation (mo/dd/year)**	08/07/2001	07/17/2002	07/29/2003	01/16/2004	01/30/ 2004	04/16/ 2004	
**Days in the ICU until the isolation of the pandrug-resistant strain**	40	62	34	33	52	32	0
**Prior colistin use (days)**	0	23	11	33	31	6	0
**Antibiotic combination used after isolation**	Colistin + imipenem/ cilastatin, Colistin + meropenem, Colistin + ofloxacin	Two days prior to the isolation, all antibiotics had been stopped	Colistin + meropenem + ofloxacin + gentamicin	Colistin + meropenem	Colistin + ampicillin/sulbactam + trimethoprim/sulfamethoxazole	Colistin + ceftazidime	Piperacillin/tazobactam
**Dosage/Duration of colistin**	2 million IU q8h iv for 35 days, 1 million IU q8h iv for 17 days	1 million IU q8h iv for 23 days	3 million IU q8h iv for 37 days, 1 million IU q8h nebulized for 11 days	2 million IU q8h iv for 16 days, 1 million IU q8h iv for 12 days, 1 million IU q12h iv for 19 days	1 million IU q8h iv for 34 days	2 million IU q8h in for 35 days, 1 million IU q8h iv for 17 days	2.25 gr q8h iv for 10 days
**Outcome of the infection**	Cure (clinical + microbiological).	No response. Patient died due to meningitis.	Cure (clinical + microbiological).	Clinical cure (microbiological persistence for 1 month).	Deterioration. Superinfection of the respiratory tract by an *Acinetobacter baumannii *strain. Patient died due to sepsis.	Cure (clinical + microbiological).	Cure (clinical + microbiological).

## Pre-publication history

The pre-publication history for this paper can be accessed here:



## References

[B1] Garnacho-Montero J, Ortiz-Leyba C, Jimenez-Jimenez FJ, Barrero-Almodovar AE, Garcia-Garmendia JL, Bernabeu-WittelI M, Gallego-Lara SL, Madrazo-Osuna J (2003). Treatment of multidrug-resistant Acinetobacter baumannii ventilator-associated pneumonia (VAP) with intravenous colistin: a comparison with imipenem-susceptible VAP. Clin Infect Dis.

[B2] Michalopoulos AS, Tsiodras S, Rellos K, Mentzelopoulos S, Falagas ME (2005). Colistin treatment in patients with ICU-acquired infections caused by multiresistant Gram-negative bacteria: the renaissance of an old antibiotic. Clin Microbiol Infect.

[B3] Hsueh PR, Teng LJ, Chen CY, Chen WH, Yu CJ, Ho SW, Luh KT (2002). Pandrug-resistant Acinetobacter baumannii causing nosocomial infections in a university hospital, Taiwan. Emerg Infect Dis.

[B4] Kuo LC, Yu CJ, Lee LN, Wang JL, Wang HC, Hsueh PR, Yang PC (2003). Clinical features of pandrug-resistant Acinetobacter baumannii bacteremia at a university hospital in Taiwan. J Formos Med Assoc.

[B5] Denton M, Kerr K, Mooney L, Keer V, Rajgopal A, Brownlee K, Arundel P, Conway S (2002). Transmission of colistin-resistant Pseudomonas aeruginosa between patients attending a pediatric cystic fibrosis center. Pediatr Pulmonol.

[B6] Tamm M, Eich C, Frei R, Gilgen S, Breitenbucher A, Mordasini C (2000). [Inhaled colistin in cystic fibrosis]. Schweiz Med Wochenschr.

[B7] Catchpole CR, Andrews JM, Brenwald N, Wise R (1997). A reassessment of the in-vitro activity of colistin sulphomethate sodium. J Antimicrob Chemother.

[B8] Manikal VM, Landman D, Saurina G, Oydna E, Lal H, Quale J (2000). Endemic carbapenem-resistant Acinetobacter species in Brooklyn, New York: citywide prevalence, interinstitutional spread, and relation to antibiotic usage. Clin Infect Dis.

[B9] Giamarellos-Bourboulis EJ, Sambatakou H, Galani I, Giamarellou H (2003). In vitro interaction of colistin and rifampin on multidrug-resistant Pseudomonas aeruginosa. J Chemother.

[B10] Pitt TL, Sparrow M, Warner M, Stefanidou M (2003). Survey of resistance of Pseudomonas aeruginosa from UK patients with cystic fibrosis to six commonly prescribed antimicrobial agents. Thorax.

[B11] Spence RP, Towner KJ, Henwood CJ, James D, Woodford N, Livermore DM (2002). Population structure and antibiotic resistance of Acinetobacter DNA group 2 and 13TU isolates from hospitals in the UK. J Med Microbiol.

[B12] Valero E, Sevillano D, Calvo A, Garcia R, Leturia A, Gomez-Lus ML (2001). [Activity of new fluoroquinolones against clinical isolates of Acinetobacter baumannii]. Rev Esp Quimioter.

[B13] Gaynes RP, Horan TC, Mayhall CG (1996). Surveillance of nosocomial infections. Appendix A: CDC definitions of nosocomial infections. Hospital epidemiology and infection control.

[B14] National Committee for Clinical Laboratory Standards (1981). Performance standards for antimicrobial disc susceptibility tests. Approved standard M2-A2 S2. Waynee, PA NCCLS.

[B15] Rynn C, Wootton M, Bowker KE, Alan HH, Reeves DS (1999). In vitro assessment of colistin's antipseudomonal antimicrobial interactions with other antibiotics. Clin Microbiol Infect.

[B16] Giamarellos-Bourboulis EJ, Karnesis L, Giamarellou H (2002). Synergy of colistin with rifampin and trimethoprim/sulfamethoxazole on multidrug-resistant Stenotrophomonas maltophilia. Diagn Microbiol Infect Dis.

[B17] Gunderson BW, Ibrahim KH, Hovde LB, Fromm TL, Reed MD, Rotschafer JC (2003). Synergistic activity of colistin and ceftazidime against multiantibiotic-resistant Pseudomonas aeruginosa in an in vitro pharmacodynamic model. Antimicrob Agents Chemother.

[B18] Ciofu O, Fussing V, Bagge N, Koch C, Hoiby N (2001). Characterization of paired mucoid/non-mucoid Pseudomonas aeruginosa isolates from Danish cystic fibrosis patients: antibiotic resistance, beta-lactamase activity and RiboPrinting. J Antimicrob Chemother.

[B19] Falagas ME, Kasiakou SK (2005). Colistin: the revival of polymyxins for the managment of multidrug-resistant Gram-negative bacterial infections. Clin Infect Dis.

[B20] Moore RA, Chan L, Hancock RE (1984). Evidence for two distinct mechanisms of resistance to polymyxin B in Pseudomonas aeruginosa. Antimicrob Agents Chemother.

[B21] Groisman EA, Kayser J, Soncini FC (1997). Regulation of polymyxin resistance and adaptation to low-Mg2+ environments. J Bacteriol.

[B22] Nicas TI, Hancock RE (1980). Outer membrane protein H1 of Pseudomonas aeruginosa: involvement in adaptive and mutational resistance to ethylenediaminetetraacetate, polymyxin B, and gentamicin. J Bacteriol.

[B23] Moore RA, Hancock RE (1986). Involvement of outer membrane of Pseudomonas cepacia in aminoglycoside and polymyxin resistance. Antimicrob Agents Chemother.

[B24] Gunn JS, Lim KB, Krueger J, Kim K, Guo L, Hackett M, Miller SI (1998). PmrA-PmrB-regulated genes necessary for 4-aminoarabinose lipid A modification and polymyxin resistance. Mol Microbiol.

[B25] Bengoechea JA, Skurnik M (2000). Temperature-regulated efflux pump/potassium antiporter system mediates resistance to cationic antimicrobial peptides in Yersinia. Mol Microbiol.

[B26] Ito-Kagawa M, Koyama Y (1980). Selective cleavage of a peptide antibiotic, colistin by colistinase. J Antibiot (Tokyo).

[B27] Gales AC, Reis AO, Jones RN (2001). Contemporary assessment of antimicrobial susceptibility testing methods for polymyxin B and colistin: review of available interpretative criteria and quality control guidelines. J Clin Microbiol.

